# Subcellular localization and function analysis of PINK1 mitron in PD progression: Mitron modulates mitochondrial morphology to regulate neuronal death

**DOI:** 10.1016/j.jbc.2024.107773

**Published:** 2024-09-12

**Authors:** Yu Qiao, Jiayuan Kou, Ye Tian, Wenkai Ma, Yang Yu, Jingjing Pang, Yingting Pei, Yu Zhang, Bin Ye, Ziying Xie, Jinying Liu, Zhihui Wang, Lujing Wang, Xu Gao, Ning Ma, Yanfen Zhang

**Affiliations:** 1 Department of Biochemistry and Molecular Biology, Harbin Medical University, Harbin, China; 2 Key Laboratory of Cardiovascular Medicine Research (Harbin Medical University), Ministry of Education, Harbin, China; 3 Translational Medicine Center of Northern China, Harbin Medical University, Harbin, China; 4 Medical Science Institute of Hei Longjiang Province, Harbin, China; 5 Department of Laboratory Diagnosis, Second Affiliated Hospital of Harbin Medical University, Harbin, China

**Keywords:** PINK1, mitochondria, ATP, Parkinson’s disease, miRNA

## Abstract

Parkinson’s disease (PD) is a multifactorial neurodegenerative disorder. Loss or degeneration of the dopaminergic neurons in the substantia nigra and development of Lewy bodies in dopaminergic neurons were the defining pathologic changes. MiRNAs fine-tune the protein levels by posttranscriptional gene regulation. MiR-7019-3p is encoded within the fifth intron of PD-associated protein PINK1. In present study, we firstly demonstrated miR-7019-3p expression is significantly upregulated in PD mice model and neuron cell models, miR-7019-3p mainly existed in mitochondria, miR-7019-3p could regulate the structure, and function of mitochondria in neuronal cells. We predicted and verified that mitochondria-associated protein optic atrophy 1 and 12s rRNA, 16s rRNA, and polycistronic RNA are target genes of miR-7019-3p. Finally, we proved that SP1 protein could independently regulate the expression of miR-7019-3p at the upstream. The evidences in the study suggest the role miR-7019-3p in the regulation of mitochondrial structure and function, and this kind of regulation could be implemented or promoted through the pathway of SP1-miR-7019-3p-optic atrophy 1/12s rRNA, 16s rRNA, and polycistronic RNA. Our results have suggested a promising and potential therapeutic target for reversing mitochondria dysregulation in neuronal cells during PD process.

Parkinson’s disease (PD) is a second most common neurodegenerative movement disorder disease. About 2% of the population above the age of 60 is affected by the disease (1). The pathological hallmarks of PD include the presence of intracytoplasmic proteinaceous retention termed as Lewy bodies and dystrophic neurites (Lewy neurites) in surviving neurons. These aggregates consist of fibrillar α-synuclein, molecular chaperones, ubiquitin, and neurofilaments (2). The temporary and selective deficiency mechanism in dopaminergic neurons during PD had become an emphasis in this research field; nevertheless, the underlying mechanism is still not well illustrated. Several therapies had been suggested for the remedy; however, supplementation of dopamine could only provide symptomatic relief. To investigate and illustrate the regulating mechanism during the neuronal excalation in PD is essential for the next generation of therapeutic strategies.

Mitochondria are found as clusters of free-floating organelles in the cytosol. The main function of mitochondria is to generate energy in the form of ATP. The structure of mitochondria which are highly dynamic is complex. Intermediate products of pyruvate oxidation and the Krebs cycle are stored in mitochondria. They also regulate calcium homeostasis, and play a role in scavenging free radicals and in controlling of programmed cell death. The matrix of the mitochondria carries 10 to 100 or more copies of a small circular mitochondrial DNA. Mitochondria were believed to be rigid organelles in the past, but present studies show that mitochondria undergo constant morphological changes by the process of continuous cycles of fusion and fission, which gives rise to mitochondria with different morphologies. The balance between fusion and fission determines most functions of mitochondria, controls its bioenergetic function, mitochondrial turnover, and protects mitochondrial DNA (3).

Defects in mitochondrial respiration are involved in PD. The first study that showed that mitochondria play a role in PD pathogenesis came from studies in which accidental infusions of the toxin 1-methyl-4-phenyl-1, 2, 3, 6-tetrahydrodropyridine (MPTP) selectively inhibited mitochondrial complex I which is one of the components of the electron transport chain (4, 5). Rotenone, pyridaben, trichloroethylene, and fenpyroximate are other inhibitors of complex I, they could induce dopaminergic neurodegeneration in flies, humans, and rodents, suggesting that mitochondrial dysfunction plays a role in PD (6). These toxins affecting mitochondria cause defects in the activity of the mitochondrial electron transport complex (7), reduce movement of mitochondria (8), cause an increase in the mitochondrial permeability transition, increase the generation of reactive oxygen species, and damage the activity of nitric oxide synthase in the mitochondria. In the substantia nigra, skeletal muscles and platelets of PD patients, the activity of complex I is impaired (6, 9, 10). After systemic administration of rotenone, mice and rats developed a robust PD phenotype (11). The catalytic subunits of complex I isolated from mitochondria in the frontal cortex of PD patients are found to be damaged due to oxidative stress and are associated with dysfunction and misassembly of complex I (12).

PINK1 (PARK6) also have mutations that can cause autosomal recessive form of Parkinsonism. This is a familial form and an early-onset PD (13). PINK1 mutations or knockdown of PINK1 lead to a decrease in respiration of mitochondria decrease in the synthesis of ATP, along with increase in the aggregation of a-synuclein in cell culture models of PD (14). Mice that lack PINK1 show reduced levels of respiration in mitochondria, along with being more susceptible to the toxic effects of oxidative stress and increased mitochondrial dysfunction (15, 16). Recent studies have shown that lowering mitochondrial membrane potential limits the import of PINK1 into the matrix mediated by its mitochondrial-targeting sequence. In depolarized mitochondria, this causes PINK1 to accumulate in the outer mitochondrial membrane (17). TOMM40 is a mitochondrial translocase that resides between the putative transmembrane domain and the mitochondrial-targeting sequence. PINK1 requires TOMM40 for localizing in the mitochondria and its phosphorylation of critical serines of ubiquitin results in Parkin recruitment, which then leads to mitophagy (18). Therefore, dysfunction of PINK1 causes defects in its localization as well as impaired mitophagy. Studies of the combination of Parkin and PINK1 KOs in *Drosophila* showed that they function in the same pathway and that PINK1 is upstream of Parkin (19). In the mitophagy pathway, PINK1 and PARKIN function together. In damaged mitochondria, accumulation of PINK1 occurs on the outer membrane and this in turn leads to recruitment of Parkin to the mitochondria which then leads to ubiquitination of proteins that are located on the outer membrane of the mitochondria to triggers selective mitophagy. Therefore, mutations in Parkin and PINK1 cause defects in function of the mitochondria and mitophagy (19).

Small noncoding RNAs, specifically miRNAs, play an important role in the regulation of mRNA copy number and protein level in the narrow physiological range (20). The role of miRNAs in regulation of different steps of autophagy and mitochondrial homeostasis and its implication in PD stress condition is not well understood. MiR-7019 is encoded in the fifth intron of mice *Pink1* gene. The function of miR-7019-5p or miR-7019-3p has not been well investigated. In this study, we firstly demonstrated the subcellular location of miR-7019-3p and function of miR-7019-3p in the regulating of mitochondria. Interestingly, we found that miR-7019-3p is significantly upregulated in induced PD mice model and the same trend in neuron cell models. Meanwhile, subcellular location results showed miR-7019-3p mainly existed in mitochondria. We demonstrated that miR-7019-3p could regulate the structure and function of mitochondria in neuronal cells. And, we predicted and verified that mitochondria-related protein optic atrophy 1 (OPA1) mRNA and mitochondrial deoxyribonucleic acid (mtDNA) encoded 12s rRNA, 16s rRNA, and polycistronic RNA could all be regulated by miR-7019-3p at transcription level. Finally, we proved that SP1 protein could regulate the expression of miR-7019-3p at the upstream of miR-7019 independently. The evidences in the study suggest the role miR-7019-3p in the regulation of mitochondrial structure and function, and this kind of regulation could be implemented or promoted through the pathway of SP-1-miR-7019-3p-OPA1/12s rRNA, 16s rRNA, and polycistronic RNA. Our results have suggested a promising and potential therapeutic target for mitochondria dysregulation in neuronal cells during PD process.

## Results

### Expressions and subcellular localization of miR-7019-3p are altered in MPTP/MPP^+^-induced PD mouse/neuron cell model

**Figure 1:**
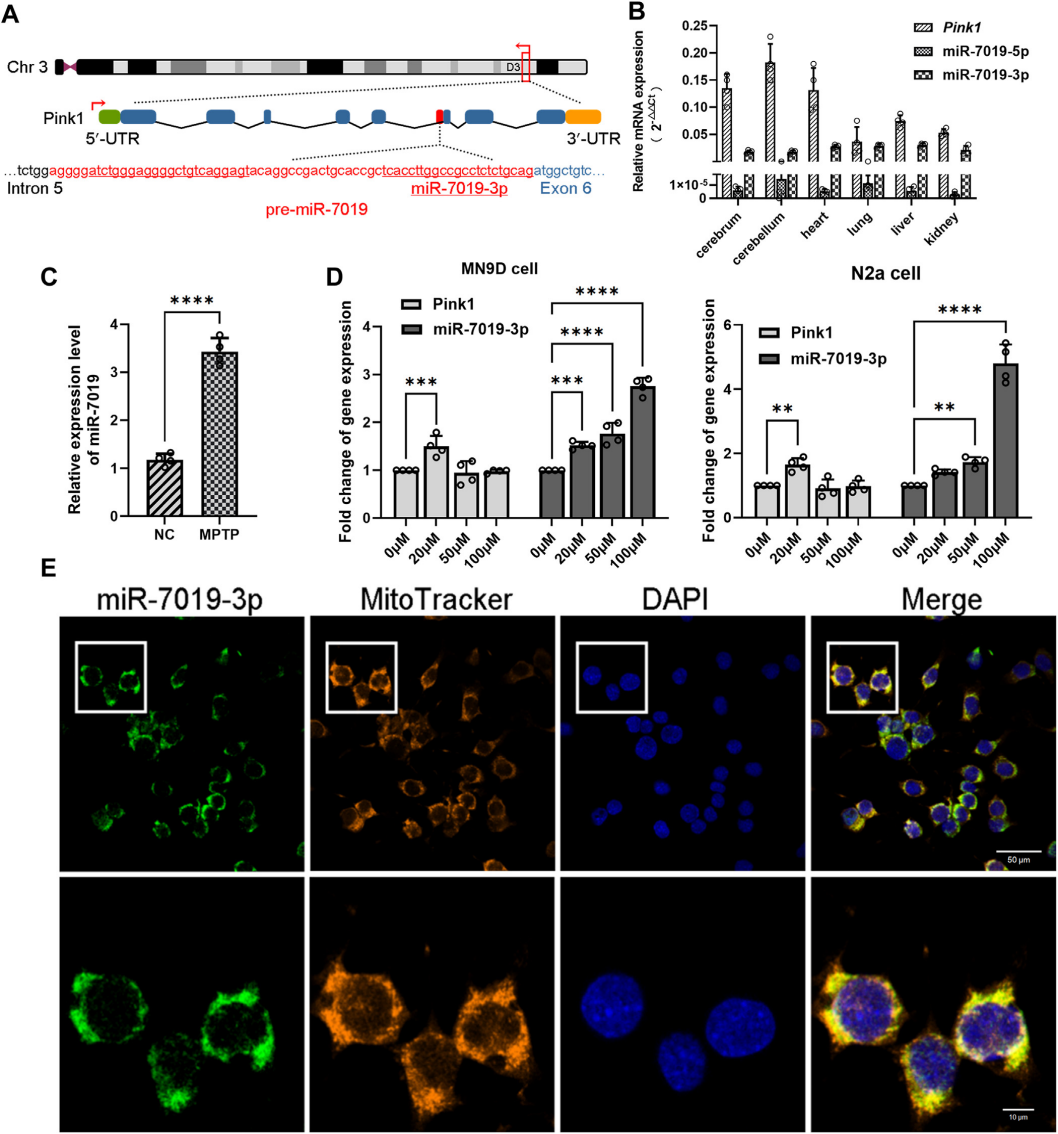
**Expressions and subcellular localization of miR-7019-3p in MPTP/MPP**^**+**^**-induced PD mouse neuron cell model**. *A*, the location of miR-7019-3p gene within mouse *Pink1* gene. *B*, expression levels of *Pink1* and miR-7019 in main organs of mouse. *C*, MiR-7019-3p expression levels in mesencephalon of MPTP-induced PD mice model and control group. *D*, *Pink1* mRNA and miR-7019-3p expression levels in MN9D cells and N2a cells. *E*, subcellular location of miR-7019-3p. *Green probe*: FISH staining of miR-7019-3p. *Red probe*: mitotracker staining of mitochondria. The scale bar represents 50 μm. ∗*p* < 0.05; ∗∗*p* < 0.01; ∗∗∗*p* < 0.001; and ∗∗∗∗*p* < 0.0001. MPTP, 1-methyl-4-phenyl-1, 2, 3, 6-tetrahydrodropyridine; MPP^+^, 1-methyl-4-phenylpyridiniumion; PD, Parkinson’s disease.

MiR-7019-3p is encoded within the fifth intron of mouse *Pink1* gene (Fig. 1*A*). To investigate the distribution of *Pink1* and miR-7019, expression levels of which are evaluated in main organs of mouse (Fig. 1*B*). The result showed that *Pink1* and miR-7019-5p/miR-7019-3p are expressed in all important organs in mouse. Meanwhile, miR-7019-3p is higher expressed in brain and other organs than miR-7019-5p. So, we mainly focused on the miR-7019-3p expression and function during mitochondria function analysis. We detected tyrosine hydroxylase in MPTP-induced PD mice model (Fig. S1*A*), to identify the PD model. In mesencephalon of MPTP-induced PD mice model, miR-7019-3p expression level was increased (Fig.1*C*). After various concentrations induction of 1-methyl-4-phenylpyridiniumion (MPP^+^) on neuron cells, PINK1 mRNA expression level was increased and miR-7019-3p was also increased, both in MN9D cells and N2a cells (Fig. 1*D*). In MPTP-induced PD mice model, the results showed that miR-7019-3p exists in cytoplasm around the cell nucleus (Fig. S1*B*). We detected subcellular localization of miR-7019-3p in MPP^+^-treated N2a cells, and the results showed that miR-7019-3p mainly existed in mitochondria; it was also shown up in cytoplasm (Fig. 1*E*). As we found that miR-7019-3p mainly existed in mitochondria, according to the previous report, we used FAM and CY3-modified synthetic miR-7019-3p to transfect into neuron cells, the fluorescence signals of exogenous transfection of miR-7019-3p were mainly found in the cytoplasm, and the fluorescence signals did not converge with the mitochondrial fluorescence signals (Fig. S1*C*).

### Overexpression of miR-7019-3p in neuron cells induced the damage to mitochondrial structure

**Figure 2:**
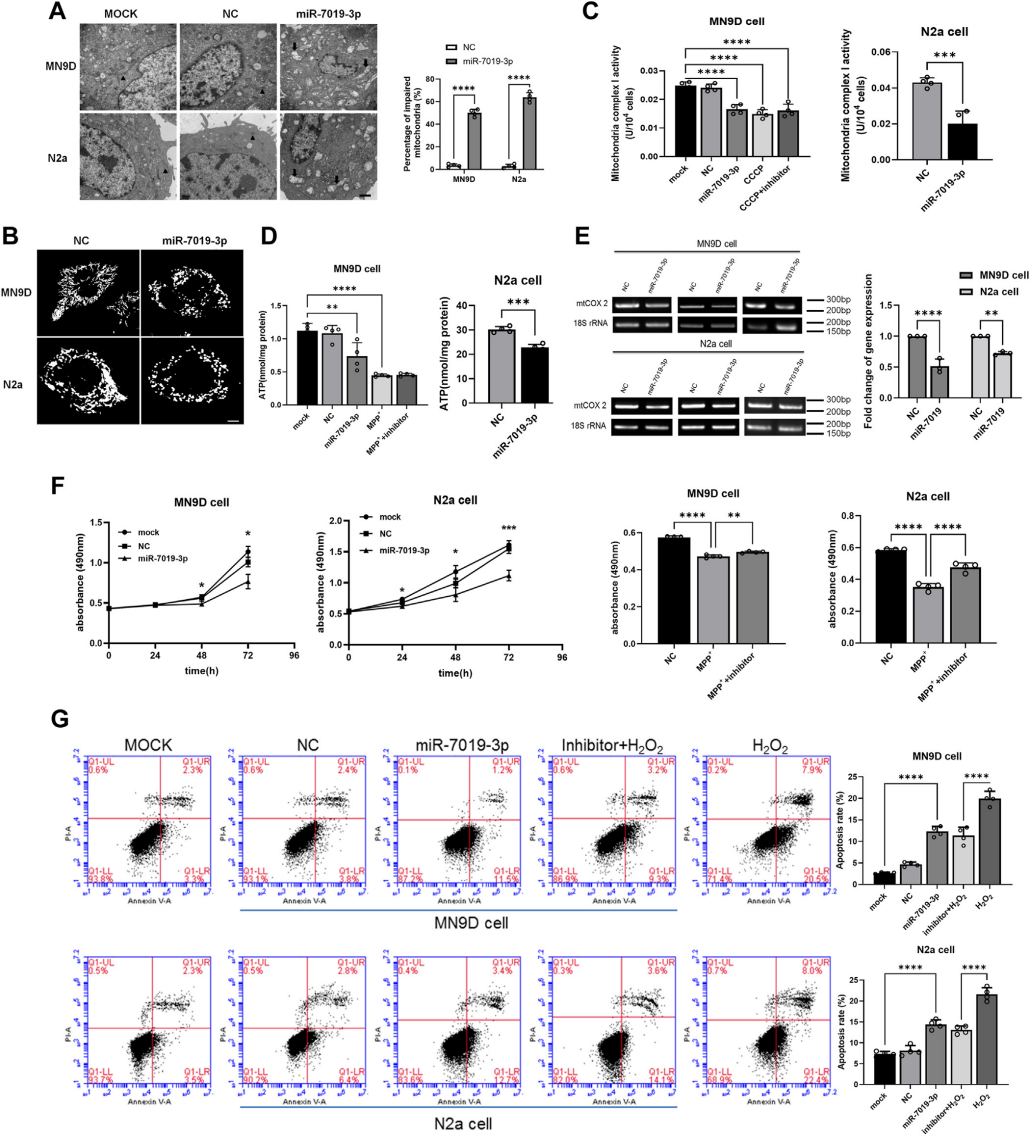
**The function of miR-7019-3p in neuron cells**. *A*, electron microscope images of mitochondrial in MN9D and N2a cells overexpressed with miR-7019-3p and control mimics. The scale bar represents 500 nm. *B*, confocal microscopy images of mitochondria, the number of point-like mitochondria increased, and the reticular structure of mitochondria was destroyed. The scale bar represents 2 μm. *C*, the activity of mitochondria complex I in MN9D and N2a cells overexpressed with miR-7019-3p is evaluated. *D*, detection of ATP content in MN9D cells overexpressed with miR-7019-3p. MiR-7019-3p expression level in N2a cells treated with MPP^+^. *E*, copy number fold change of the mitochondrial DNA. *F*, MTT experiment results of cells overexpressed and inhibition with miR-7019-3p. *G*, flow cytometry results to detect the apoptosis of cells transfected with miR-7019-3p mimics or inhibitors. ∗*p* < 0.05; ∗∗*p* < 0.01; ∗∗∗*p* < 0.001; and ∗∗∗∗*p* < 0.0001. MPP^+^, 1-methyl-4-phenylpyridiniumion; MTT, methyl thiazolyl tetrazolium.

We would like to investigate the function in cytoplasm for the further research, so we overexpressed and inhibited miR-7019-3p in neuron cells. Electron microscope result showed overexpression of miR-7019-3p lead to increasing mitochondrial swelling and decreasing distinct mitochondrial cristae, resulting damage of mitochondria in MN9D and N2a cells (Fig. 2*A*). Mitochondrial dynamics is another important indicator for the status of mitochondria. When mitochondrial fusion increased, mitochondria mainly showed long tubular or reticular structure, while mitochondria with increased division mainly showed dot structure. We transfected miR-7019-3p mimics in MN9D and N2a cells, staining the living cells with MitoTracker, and observing the morphology of the lineaments with confocal microscopy. After overexpression of miR-7019-3p in MN9D and N2a cells, the number of point-like mitochondria increased and the reticular structure of mitochondria was destroyed (Fig. 2*B*). The result indicated that overexpression of miR-7019-3p in neuron cells could accelerate fission of mitochondria. The activity of mitochondria complex I is also monitored after transfection with miR-7019-3p mimics in MN9D and N2a cells. Overexpression of miR-7019-3p could inhibit the activity of mitochondria complex I in MN9D and N2a cells (Fig. 2*C*). After overexpression of miR-7019-3p in cells, ATP levels in N2a and MN9D cells significantly decreased, and MPP^+^ treatment significantly reduced ATP levels in MN9D cells (Fig. 2*D*). To observe the changes in the copy number of mitochondrial DNA, after overexpressing miR-7019-3p in MN9D and N2a cells, we extracted the total DNA of the cells. We selected the nuclear gene 18S rRNA as the internal reference gene to observe the expression of mitochondrial gene COX2, to detect the copy number fold change of the mitochondrial DNA(Fig. 2*E*). Methyl thiazolyl tetrazolium experiment results showed that overexpression of miR-7019-3p in MN9D and N2a cells could decrease the cell viability, meanwhile, inhibition of miR-7019-3p expression can alleviate the damage of the cells (Fig. 2*F*). Mitochondria could regulate the process of apoptosis. When apoptosis occurs, some proapoptotic proteins can be transferred to mitochondria to damage the permeability and integrity of mitochondrial membrane and then release apoptosis induction factors to mediate apoptosis. When mitochondrial function is damaged, changes of mitochondrial membrane permeability can also cause apoptosis. It has also been reported that increased mitochondrial fission can cause apoptosis. MiR-7019-3p can not only promote mitochondrial fission but also reduce cell activity, so we used flow cytometry to detect the apoptosis of cells transfected with miR-7019-3p mimics or inhibitors. We found that overexpression of miR-7019-3p can induce apoptosis of cells, and inhibition of miR-7019-3p expression can alleviate H_2_O_2_-induced apoptosis (Fig. 2*G*).

### MiR-7019-3p targets mitochondria-related genes as a regulator

**Figure 3:**
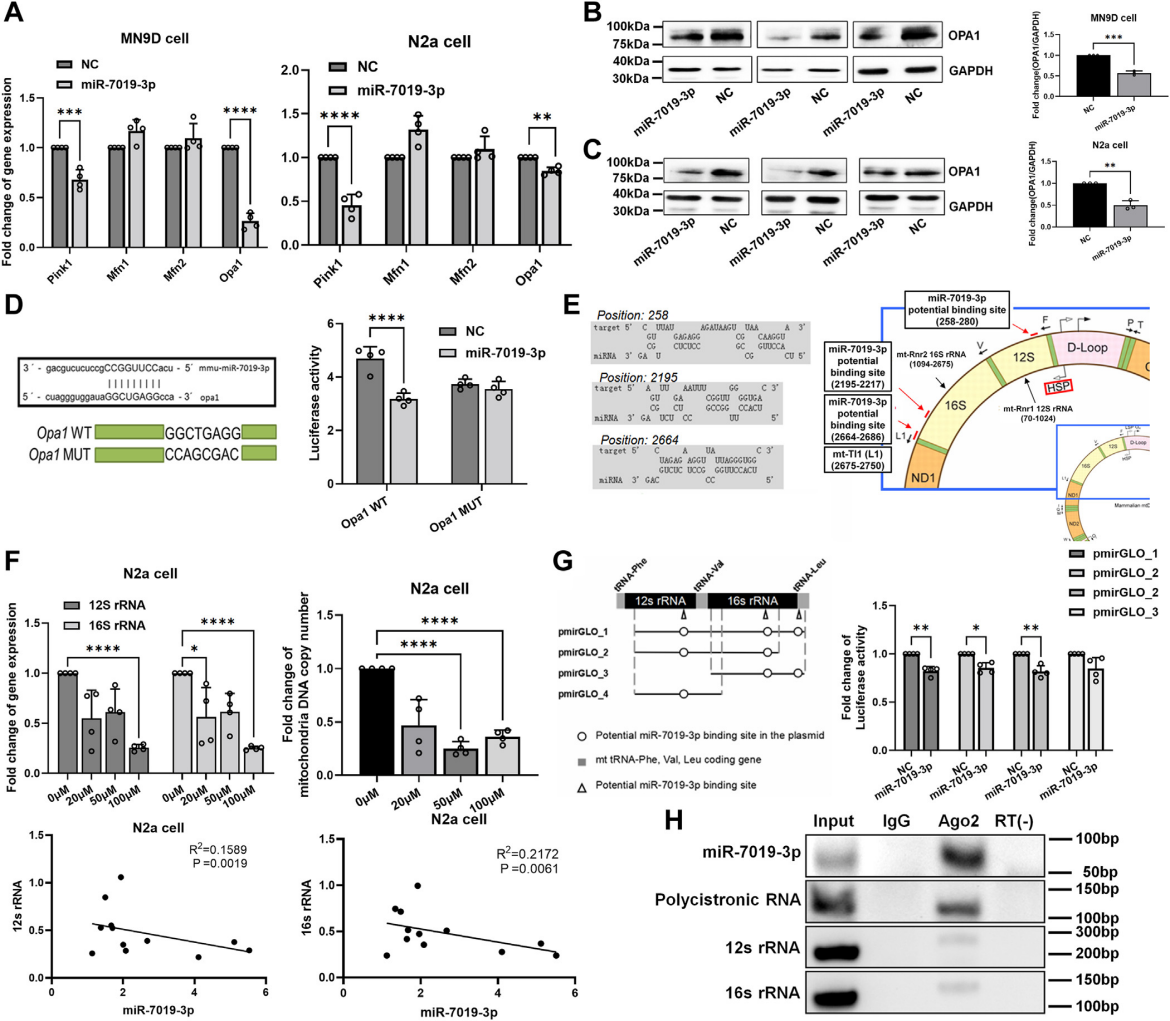
**Prediction and verification of miR-7019-3p′s target genes**. *A*, expression level of miR-7019-3p–targeting genes in cells overexpressed with miR-7019-3p. *B*, expression level of OPA1 protein in MN9D cells. *C*, expression level of OPA1 protein in N2a cells. *D*, binding region of miR-7019-3p in mRNA 3′ UTR of *opa1*. Luciferase activity detection of opa1 WT and MUT groups. *E*, the mitochondria encoded RNA 12s rRNA, 16s rRNA, and polycistronic RNA were predicted as target genes of miR-7019-3p. Potential binding sites between miR-7019-3p and targeting RNAs above. *F*, expression level of 12s and 16s rRNAs and copy number of mitochondria DNA after treated with MPP^+^ in N2a cells. Pearson correlation coefficients between miR-7019-3p and 12s rRNA and 16s rRNA. *G*, after cotransfection of miR-7019-3p and pmirGLO plasmid carried with kinds of target mRNA groups. *H*, RIP experiment of mitochondrial components including Ago2 protein, to detect Ago2 binding miR-7019-3p and mitochondrial long RNAs. ∗*p* < 0.05; ∗∗*p* < 0.01; ∗∗∗*p* < 0.001; and ∗∗∗∗*p* < 0.0001. Opa 1, optic atrophy 1. MPP^+^, 1-methyl-4-phenylpyridiniumion; RIP, RNA binding protein immunoprecipitation.

The result showed that miR-7019-3p could regulate the function of mitochondria, and the subcellular localization of which is mainly in mitochondria and cytosol of neuron cells. We further used RNAhybrid on line to predict and select the target genes of miR-7019-3p in mitochondria morphology and function. In the cytoplasm level, we found pink1, mfn1, mfn2, and opa1 as target genes of miR-7019-3p, after the detection of mRNA level of above genes, we found that overexpression of miR-7019-3p could obviously decrease opa1 mRNA expression level (Fig. 3*A*). OPA1 is an important protein that regulates mitochondrial intimal fusion. In addition, OPA1 can also regulate the remodeling of mitochondrial cristae, thus affecting the apoptosis of mitochondria. After overexpression of miR-7019-3p, the expression level of OPA1 protein or opa1 mRNA was significantly downregulated compared with that of the control group in MN9D cells (Fig. 3*B*). Based on the above results, miR-7019-3p can inhibit the expression of Opa1 from both transcription and translation levels in N2a cells (Fig. 3*C*). To test if miR-7019-3p directly binds to the 3′UTR of opa1 mRNA, luciferase reporter system experiment was conducted. The results showed that compared with the control group, luciferase activity was significantly decreased when miR-7019-3p mimics and the opa1-binding sequence WT plasmids were transfected simultaneously (Fig. 3*D*). The luciferase activity of miR-7019-3p mimics and the opa1-binding sequence mutant plasmid groups was not significantly changed.

In mitochondria, due to mtDNA gene expression process, promoter H1 begins transcription and produces precursor RNA molecule containing 12s rRNA, 16s rRNA, and the precursor of them, which is called polycistronic RNA. We found 12s rRNA, 16s rRNA, and polycistronic RNA were predicted as target genes of miR-7019-3p (Fig. 3*E*). According to the above predicted results, the expressions of 12s rRNA and 16s rRNA in N2a cells treated with MPP^+^ at different concentrations were detected in this study, and the results showed that their decreased expressions (Fig. 3*F*). Through correlation analysis of miR-7019-3p with 12s rRNA and 16s rRNA (Pearson correlation analysis), the content of miR-7019-3p is negatively correlated with the level of 12S rRNA and 16S rRNA (Pearson correlation coefficients are −0.4298 and −0.4638, respectively) (Fig. 3*F*). We used pmirGLO plasmid as Dual Luciferase Reporter system to verify the binding of miR-7019-3p and the target gene mRNAs. There were all three potential binding sites of genes on pmirGLO-1, and 12s rRNA together with 16s rRNA potential binding sites on pmirGLO-2, and 16s rRNA together with 16s tRNA^Leu^ parts potential binding sites on pmirGLO-3, and only potential binding sites of the 12s rRNA on pmirGLO-4(Fig. 3*G*). The Dual Luciferase Reporter experiment results showed that compared with the control of mimics negative control, the luciferase activities of overexpressed miR-7019-3p in the pmiRGLO-1, pmiRGLO-2, and pmiRGLO-3 groups were significantly decreasing, suggesting that miR-7019-3p bind effectively with the above predicted sequences (Fig. 3*G*). To further confirm that miR-7019-3p could target and binding to the mitochondrial rRNA and its precursor polycistronic RNA directly, the RNA binding protein immunoprecipitation (RIP) experiment of mitochondrial components including Ago2 protein was performed. Mitochondria were isolated, and Ago2 complex was captured by Ago2 antigen-magnetic beads, Ago2-binding miR-7019-3p, and mitochondrial long RNAs were then detected. The results shows that miR-7019-3p could bind to 12S rRNA, 16S rRNA, and polycistronic RNA through Ago2 protein, the interaction between miR-7019-3p and the predicted target RNAs were suggested (Fig. 3*H*).

### Regulation of miR-7019-3p transcription by Sp1 within pink1 gene

**Figure 4:**
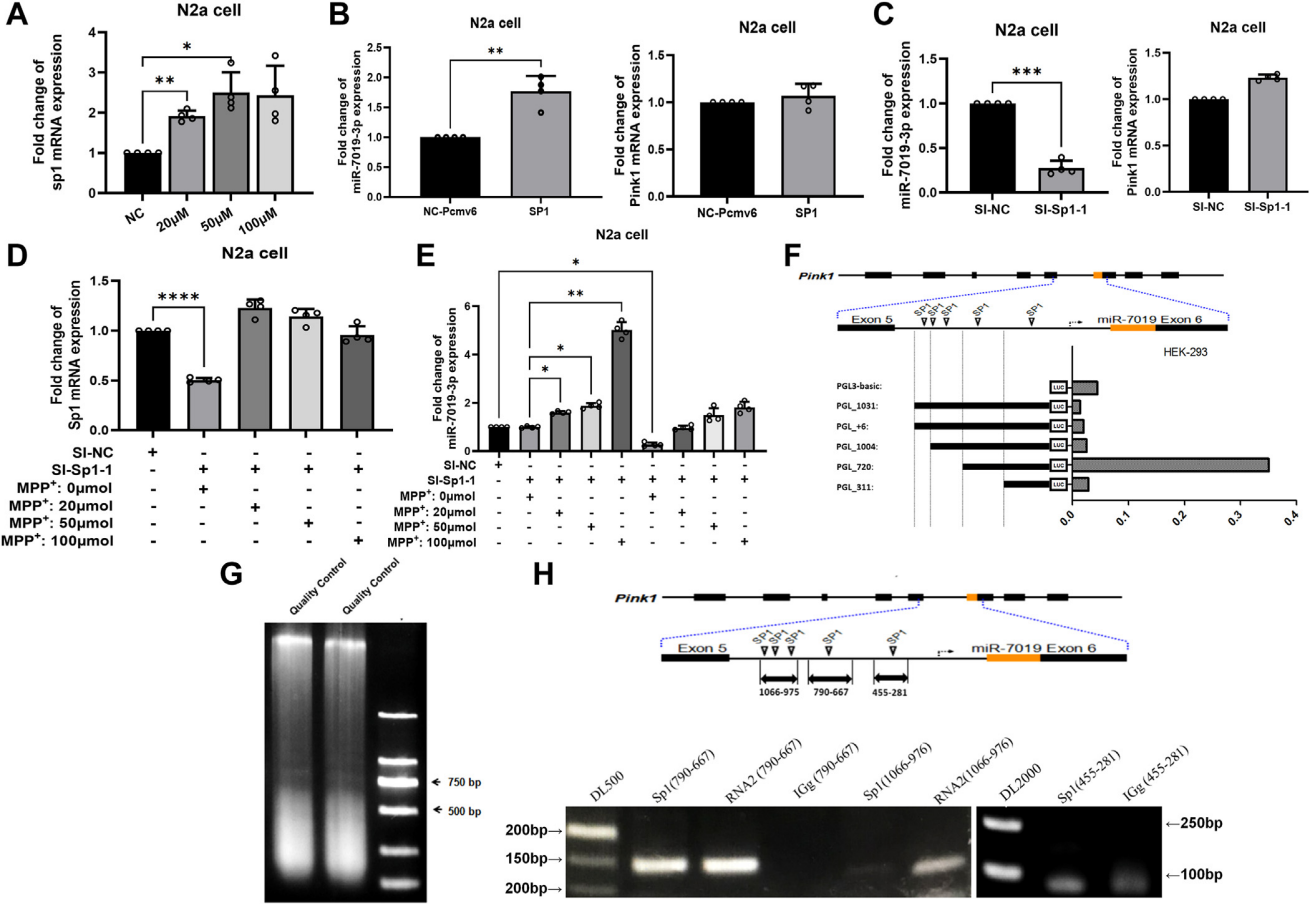
**Prediction and verification of miR-7019-3p′s transcriptional regulatory protein**. *A*, Sp1 mRNA expression level in N2a cells treated with MPP^+^. *B*, expression level of miR-7019-3p and *pink1* in N2a cells overexpressed with *sp1* protein. *C*, expression level of miR-7019-3p and *pink1* mRNA in N2a cells treated with siRNA of *sp1*. *D*, expression level of *Sp1* mRNA in N2a cells treated with siRNA of *sp1* mRNA and different concentration of MPP^+^. *E*, expression level of miR-7019-3p in N2a cells treated with siRNA of *sp1* mRNA and different concentration of MPP^+^. *F*, five kinds of chimeric plasmids based on PGL3 basic plasmid, which carried with different types of sheared binding region sequences of SP1 on upstream of miR-7019 encoded region. The activities of luciferase reporter gene product were detected among six kinds of PGL3 basic plasmids, to show the activated region of SP1. *G* and *H*, ChIP result to verify the binding of Sp1 protein and miR-7019-3p upstream DNA sequence. ∗*p* < 0.05; ∗∗*p* < 0.01; ∗∗∗*p* < 0.001; and ∗∗∗∗*p* < 0.0001. ChIP, chromatin immunoprecipitation; MPP^+^, 1-methyl-4-phenylpyridiniumion.

MiR-7019-3p is encoded within the fifth intron of mouse *pink1* gene. As we found that after the MPP^+^ treatment of the neuron cells, increasing proportion of miR-7019 is higher than which of *pink1* mRNA expression (Fig. 1*D*). We would like to investigate whether the promoter of miR-7019-3p exists in the fifth intron of mouse *pink1* gene sequence. SP1 is a kind of transcription factor. We predicted the binding sequence of SP1 on the upstream of miR-7019-3p encoded region (Fig. S2). As shown in the figure, a total of 425 binding sites of transcription factors were found in the positive and antisense chains, and 34 predictive sites on the justice chain (generally referred to as the coding chain) were able to bind 14 transcription factors, respectively. These transcription factors are 3′-enhancer, adipocyte protein 2), bradykinin receptor B1, BOX_4 (high mobility group protein B4), CACCC-BINDING_F, F2F, GATA-1 (GATA-binding factor 1)，GATA-2 (GATA-binding factor 2), glucocorticoid receptor，H2TF-2，MUEBP-C2，neurofibromatosis type I，SP1 (transcription factor specificity protein 1), and TATA box-binding protein. According to the previous studies, we design experiments to verify that SP1 could regulate miR-7019-3p and Pink1 mRNA expression. It has been reported that MPP^+^ drug can affect the gene expression of transcription factor SP1 (21), and the expression of transcription factor SP1 is upregulated after MPP^+^ drug treatment of human microvascular endothelial cells (22). We treated mouse neuroblastoma N2a cells with different concentrations of MPP^+^ and SP1 mRNA expression level is increasing in the cells (Fig. 4*A*), which is the same trend to *pink1* mRNA and miR-7019-3p. Then, we overexpressed *Sp1* in N2a cells (Fig. S3). The results showed that miR-7019-3p was increasing in N2a cells overexpressed with SP1 protein, meanwhile *pink1* mRNA was not significantly changed (Fig. 4*B*). Then, we used siRNA to knock down *Sp1* mRNA level in N2a cells (Fig. S4). The results showed that miR-7019-3p was downregulated in N2a cells after the knock down of SP1 mRNA, meanwhile, there was no obvious change of *pink1* mRNA expression in N2a cells (Fig. 4*C*). We used siRNA to knock down the mRNA level of Sp1, and then used MPP^+^ to stimulate the expression of SP1, we found that MPP^+^ treatment reverse the expression level of *Sp1* mRNA in N2a cells (Fig. 4*D*). The results also showed that miR-7019-3p expression was downregulated after the knock down of SP1, although MPP^+^ could stimulate the expression level of miR-7019-3p (Fig. 4*E*). To investigate the sequence within the *Pink1* gene binding to SP1 protein, we constructed WT and mutant binding sequence of miR-7019-3p to SP1 by using luciferase reporter system plasmid. As we predicted, there were five binding regions for SP1 protein located in the upstream of miR-7019, we recombined five kinds of chimeric plasmids with different types of sheared binding region sequences (Fig. 4*F*). HEK-293 cells were transfected with plasmids containing the upstream transcription factor binding sites of miR-7019, and the transcriptional activity of each predicted site at the upstream of miR-7019 gene sequence was detected among −1031 bp ∼ −1004 bp ∼ −998 bp ∼ −720 bp  ∼ −311 bp. As results shown, the luciferase reporter gene expression was very low after transfection with the PGL3-basic plasmid, and the sequence management expression of the plasmid PGL_720 containing the binding sites at −720 bp and −311 bp upstream of miR-7019 was relatively high (Fig. 4*F*). When the binding site at −720 bp upstream of miR-7019 was removed, the luciferase activity was significantly decreased, indicating that there was high transcriptional activity at −720 bp upstream of miR-7019. Then, we performed chromatin immunoprecipitation experiment to verify the binding of SP1 protein and miR-7019-3p upstream DNA sequence (Fig. 4, *G* and *H*). The results showed that the sequence containing the predicted binding site which was at −720 bp upstream of miR-7019 was detected. The sequences containing the first three predicted binding sites (upstream of miR-7019 at −1031 bp  ∼ −1004 bp ∼ −998 bp) were slightly detected. The sequence containing the fifth predicted binding site (upstream of miR-7019 at −311 bp) was slightly detected. Our study proved that SP1 transcription factor could bind to the upstream sequence of miR-7019, and the main binding site was at −720 bp upstream of miR-7019 gene.

## Discussion

Short RNAs guiding argonaute proteins regulate conserved posttranscriptional genes through diverse pathways. Among argonaute-mediated small RNA pathways, the best studied are the miRNAs (23). Generally speaking, miRNAs are 21 to 24 nt RNAs whose termini are precisely defined and derive from precursor transcripts bearing one or more inverted repeats or hairpins (24). The most prevalent alternative pathway involves short hairpin introns known as mirtrons that serve as pre-miRNA mimics (25). Mirtrons have been studied in Drosophila (25), and they also exist in *Caenorhabditis elegans* (25, 26, 27, 28) and vertebrates (29, 30, 31, 32, 33). In addition to conventional mirtrons, where splicing defines both ends of the pre-miRNA hairpin, so-called ‘‘tailed’’ mirtrons contain an unstructured region 5′ or 3′ to a splicing-derived terminal intron hairpin. Several 3′-tailed mirtrons exist in Drosophila (34). In contrast, tailed mirtrons in vertebrates are essentially only of the 5′-tailed class (30, 31, 32, 33, 34, 35), the relevant expression characteristic of mirtron and their host gene is not known.

Although mirtrons are expressed at lower levels than typical canonical miRNAs, their regulatory influence has been gaining attention. Mmu-miR-7019-3p, regarded as a 5′ tailed mirtron, derives from the fifth intron of the *Pink1* gene of mice. In previous studies on the function of *Pink1* gene, especially in mice, researchers had not demonstrated the function of mmu-miR-7019-3p. In our study, we confirmed the expression of mmu-miR-7019-3p in N2a cells and MN9D cells. We also found that after N2a cells and MN9D cells were treated with lipopolysaccharide and MPP^+^, mimicking condition of inflammation, fold changes of mmu-miR-7019-3p and its host gene *Pink1* mRNA expression are not synchronized, suggesting that in mouse dopaminergic cells, there may be an independent transcriptional regulation mechanism during mmu-miR-7019-3p expression pathway. Software analysis of putative transcription factor binding sites suggests that the 1056 bp 5′ flanking region of the mmu-miR-7019 gene contains several putative regulatory elements, including SP1 and other transcription factors. After overexpression of *Sp1* in N2a cells and MN9D cells, expression of *Pink1* mRNA and protein is increasing *in vitro*. A series of nested deletions of the 5′ flanking region fragments subcloned into the luciferase reporter plasmid pGL3-basic and assayed for luciferase activity revealed that the fifth intron of *Pink1* gene, containing the transcription initiation site, exhibits basal transcription activity of mmu-miR-7019-3p, but lacks some important regulatory elements for transcription of the *Pink1* gene. These results clearly show that the 5′ flanking region 784 bp to 790 bp sequence of mmu-miR-7019 gene contains functional SP1 response elements that regulate mmu-miR-7019-3p levels through gene expression control. SP1 plays an important role in regulating the expression of many genes. Our study indicates mmu-miR-7019 gene expression could be independently regulated by SP1 at the transcriptional level.

Subcellular distribution relationship between some intron miRNAs and their host genes has been reported. As expressed by the host gene of mmu-miR-7019-3p, PINK1 protein is in both mitochondria and cytoplasm. PINK1 protein could be cut and then transported into mitochondrial inner membrane to function. We further studied the subcellular localization of mmu-miR-7019-3p compared with PINK1 protein location. In present work, mmu-miR-7019-3p is found located both in the mitochondria and cytoplasm. The nature spontaneous mmu-miR-7019-3p is found existing in mitochondria, and the synthetical mimic mmu-miR-7019-3p forced into cells is found mainly in cytoplasm. These results indicate the processing and mature mechanisms of mmu-miR-7019-3p remain to be further discovered. There are still limited details about the subcellular distribution of mmu-miR-7019-3p that have been reported, especially in the mitochondria which are related with PINK1 protein function. Our study observed the subcellular distribution of the mmu-miR-7019-3p in neuron cells for the first time.

Pink1 is an important PD-associated gene; it has been studied in NSCs, progenitor cells and induced pluripotent stem cells, demonstrating a role for PINK1 in stem/progenitor cell proliferation and maintenance (36). MiR-24-3p is reported to target PINK1 in the PD models, leading to impaired mitophagy and neuronal damage. And circEPS15 could be protected against PD pathology through recovering PINK1-PRKN–mediated mitophagy, through sponging miR-24-3p (37). MiR-593-5p inhibits a signaling pathway involving PINK1 and Parkin, two proteins responsible for the removal of damaged mitochondria from cells, by targeting the coding sequence of PINK1 mRNA (38). During asthma process, miR-423 downregulated the expression of interleukin-1β/NOD-like receptor thermal protein domain associated protein 3/Caspase-1 inflammasome signaling by targeting PINK1 in lung cells (39). Expression of PINK1, miR-124, and miR-506 showed high impact probability in colorectal cancer (40).

In analysis of mmu-miR-7019-3p and it host gene *Pink1* biological function, our study mainly focused on the mitochondria morphology and function regulation. PINK1 has recently been shown to interact with and phosphorylate the embryonic ectoderm development polycomb histone-methylation modulator, redistributing it to the mitochondria, resulting in a favorable change in transcription regulation for neuronal differentiation (41). PINK1 is proved to be one of improving mitochondrial bioenergetics and antioxidative treatment strategies (42). Our study demonstrated miR-7019-3p exacerbated the motor and sensory functional deficits and inhibited the fusion of mitochondria. The mitochondria act at the core of the apoptotic pathway by providing many important factors including those that induce caspase activation and chromosome fragmentation (43). Disrupting mitochondrial processes may lead to the development of many diseases such as PD, a neurodegenerative disease. Given their high energy demands, neurons are cells that are most sensitive to mitochondrial damage (44, 45). In the present work, when neuron cells are treated with low concentration, PINK1 mRNA level is upregulated, the mirtron of PINK1 miR-7019-3p expression level is not significant changed; when treated with high concentration of MPP^+^, PINK1 mRNA level is not significantly changed, miR-7019-3p is obviously upregulated, and the mechanisms underlying need further investigation. We observed that overexpression of mmu-miR-7019-3p increased the apoptosis of N2a and MN9D cells and inhibition of mmu-miR-7019-3p decreased the apoptosis. Further analysis of mmu-miR-7019-3p in our study suggested its important role in PD conditions such as mitochondrial quality control in neuron cell lines. In our present work, the overexpression of mmu-miR-7019-3p in cells can cause mitochondrial swelling, decreased the number of mitochondrial cristae, increased mitochondrial fission, decreased activity, and reduced productivity of mitochondrial complex I.

The number and morphology of mitochondria are precisely controlled through mitochondrial fusion and fission machinery by mitochondria-shaping proteins (46, 47), including the large GTPases mitofusins, Mfn1 and Mfn2 (48) and Opa1 protein (49). Opa1 promotes fusion, through effects on the inner mitochondrial membrane (50, 51). The regulation of Opa1 is complex and is dependent upon cleavage at both the mRNA transcript and the protein levels (52). In our present work, we predicted that Opa1 was one of mmu-miR-7019-3p target genes. Overexpression of mmu-miR-7019-3p decreased Opa1 mRNA and protein expression level in MN9D and N2a cells. It is also demonstrated that mmu-miR-7019-3p binded to the 3′UTR region directly. Our results showed that mmu-miR-7019-3p regulated the morphology and function through target gene Opa1.

In present study, we found that endogenous mature mmu-miR-7019-3p is mainly distributed in mitochondria in N2a cells and MN9D cells. We further predicted that the potential target genes of miR-7019-3p through RNAhybrid software were 12s rRNA, 16s rRNA, and multicistronic RNA, which are generated from mtDNA. After the treatment of cells with different concentrations of MPP^+^, the expression levels of 12s rRNA and 16s rRNA decreased, and miR-7019-3p expression level increased, meanwhile, the copy number of mtDNA decreased, but the decrease fold changes were less than that of 12s rRNA and 16s rRNA. Furthermore, we examined the binding between mmu-miR-7019-3p and target genes in mitochondria. Overexpression of miR-7019-3p in cells could directly downregulate the luciferase reporter plasmid products, which contain 16s rRNA and multicistronic RNA-binding sites. Ago2 RIP experiment confirmed that Ago2 protein can bind to mir-7019-3p, 12s rRNA, 16s rRNA, multicistronic RNA, and other molecules. Most miRNAs were reported targeting the 3′-UTR region of encoding protein genes mRNAs, and our study demonstrated that miR-7019-3p could combine with the middle and last one-third region of mitochondrial 16s rRNA. In eukaryotic cells, mitochondrial ribosomes are responsible for protein synthesis within mitochondria, which are the organelles responsible for energy conversion and ATP production. Mitochondrial ribosomes have evolved specifically to synthesize mitochondrial membrane proteins. Mitochondrial ribosomes are composed of large subunits and small subunits. The sedimentation coefficient of large subunits is 39S, including 16s rRNA. The sedimentation coefficient of the small subunit is 28S, including 12s rRNA (53). In previous studies, only the mitochondrial genome-encoded hsa-miR-mit-3, hsa-miR-mit-4, and nuclear genome-encoded miR-4485 were found to be able to downregulate the expression of MT-RNR2 (16s rRNA) genes (54). In our study, another kind of miRNAs that can target mitochondrial rRNA were revealed. It also reveals the interaction between intracellular miRNAs and other noncoding RNAs from another aspect.

## Experimental procedures

### Cells and reagents

Cell culture medium is added with 10% fetal bovine serum and 1% penicillin/streptomycin, culture conditions for 37 °C and 5% CO_2_ incubator and saturated humidity. MN9D cells were cultured with Roswell Park Memorial Institute 1640 medium, 293T cells were cultured with Dulbecco's modified Eagle medium, and N2a cells were cultured with Eagle’s Minimum Essential Medium.

### RNA isolation and real-time PCR

Total RNA was extracted using TRIzol reagent (Invitrogen) in accordance with the manufacturer’s protocol. Reverse transcription of RNA was performed with high-capacity complementary DNA reverse transcription kit (ABI). Real-Time PCR was performed on a 7500 fast real-time PCR system (ABI).

### Antibodies and Western blotting

Total protein samples from cells and tissues were extracted by radio immunoprecipitation assay protein lysate (Beyotime) and were separated by SDS-PAGE, followed by blotting on a polyvinylidene fluoride film. The polyvinylidene fluoride membrane was incubated with primary antibody, followed by incubation with secondary antibody conjugated to peroxidase. The samples were analyzed for the level of GAPDH, OPA1, or NF-kB (antibodies purchased from Cell Signal Technology). Image J software (https://imagej.net/ij/) was used for quantifying protein bands by absorbance.

### Mitochondrial staining

Mitochondrial dyestuff was prepared and incubated within a lucifugal 1.5 ml tube, mitochondrial dyestuff storage solution (1 mM) 1 μl+1 ml serum-free double antibody medium, incubated in a incubator for at least 20 min. Cells were incubated with mitochondrial dye under 37 °C and in 5% CO_2_ incubator for 45 min. After staining, the cells were washed with serum-free double antibody medium 5 min for three times. Confocal microscopy was used to take pictures or continued to perform FISH.

### Fluorescent *in situ* hybridization

According to the instructions of miR-7019-3p *in situ* hybridization kit (bod, MK10201), steps are as follows: Wash the sample with PBS for three times; 4% paraformaldehyde at room temperature for 20 min, wash it with distilled water for three times; treated with 30%H_2_O_2_+ methanol (1:50) at room temperature for 30 min, washed with distilled water was washed three times; digested with pepsin at room temperature for 10s (1 ml 3% citric acid solution +2 drops pepsin); wash the sample in PBS for 5 min × 3 times with distilled water; after fixation, fixed with 1% paraformaldehyde at room temperature for 10 min, and washed with distilled water for three times; each sample was treated with 20 μl hybrid liquid under 40 °C for 2.5 h; suck out redundant prehybrid liquid, each sample was treated with 20 μl hybrid liquid containg miR-7019-3p probes, under 40 °C, avoid light and overnight incubation; washed with 2x saline sodium citrate buffer (SSC), 0.5x SSC, and 0.2x SSC under 37 °C for 20 min; 37 °C, 2x SSC, 5 min × 2 times; 37 °C, 0.5x SSC, 15 min; 37 °C, 0.2x SSC, 15 min; blocking buffer was added, incubation under 37 °C for 30 min; excess liquid was wiped off, treated with biotin rat antibody, 37 °C for 60 min; washed with PBS for 5 min × 4 times; streptavidin biotin complex-FITC (1 μl concentrate+100 μl PBS) 50 μl, 37 °C for 30 min; PBS 5 min × 3 times; treated with 4′,6-diamidino-2-phenylindole at room temperature for 4 min; washed with PBS for 5 min × 3 times, sample was sealed, and confocal images were taken.

### Intracellular ATP detection

According to the instructions of ATP detection kit (Biyuntian, S0026), the procedures are as follows: discard the culture medium and wash cells twice with PBS; cracking solution was then added; 4 °C, 12000*g* centrifuge for 10 min, liquid supernatant was transferred into a new tube; 100 μl ATP detection working fluid was still at room temperature for 5 min, then mix it with 80 μl of supernatant in the tube, and measure fluorescence value by luminometer.

### The activity detection of mitochondrial complex I

Perform according to the instruction: collect 5 × 10^6^ cells, add 1 ml reagent A and 10 μl reagent C, and homogenate them on ice; centrifuge the homogenate at 4 °C, 600*g* for 5 min; transfered the supernatant into a new centrifuge tube, centrifuge with 11,000*g*, 4 °C for 10 min; discard the supernatant, add 200 μl reagent B and 2 μl reagent C, ultrasonic crushing (ice bath, power 20% or 200 W, ultrasound for 3s, 10 s interval, repeat 30 times), for complex I enzyme activity determination; preheat the enzyme label instrument for more than 30 min, adjust the wavelength to 340 nm, and adjust to zero with distilled water; incubate working liquid at 37 °C for 5 min, each tube of 96 orifice plate add 10 μl sample, 200 μl working liquid and 15 μl reagent F, blending, recorded at 340 nm initial absorbance value A1, and absorbance values after 2 min A2, ΔA = A1-A2. Complex Ⅰ activity (U/10^4^ cells) = [ΔA × V _total action_ ÷ (ε × d) × 10^9^] ÷ (500 × V _sample_ ÷ V _total sample_) ÷ T = 1.46 × ΔAV.

### Immunoprecipitation of Ago2–RNA complexes

The centrifugal tube used for the final step of preparation of magnetic beads is placed on the magnetic rack. After the magnetic beads are attracted to the tube wall, they are carefully sucked up and removed. Nine hundred microliters RIP immunoprecipitation buffer is added to each tube. Rapid melting RIP lysate was centrifuged at 14,000 rpm at 4 °C for 10 min. One hundred microliters supernatant is added into the RIP immunoprecipitation buffer containing beads–antibody complex. The final volume of immunoprecipitation reaction is 1 ml. Then, take the supernatant of 10 μl RIP lysate into the new centrifugal tube, denoted as "input," and store the input at −80 °C. Ago2 protein was detected by supernatant of 10 μl RIP lysate, 10 μl 2 × SDS-PAGE loading buffer was added and heated at 95 °C. RIP lysate is directly available for SDS-PAGE. Incubate two centrifuge tubes at 4 °C overnight. After the centrifugal tube instantaneous away, put on the magnetic rack, carefully suction up to clear. Remove the centrifugal tube from the magnetic rack and add 500 μl RIP wash buffer to each tube for simple vortex. After instantaneous centrifugation of the centrifugal tube, put the centrifugal tube on the magnetic rack, carefully absorb the supernatant. Remove the centrifugal tube from the magnetic rack, add 500 μl RIP wash buffer into each tube, simply vortex, instantaneous away from the centrifugal tube, and then put on the magnetic rack, carefully suction on the cleaning process for five times to wash magnetic beads, each time use 500 μl precooled RIP wash buffer. In the last elution process, 50 μl from 500 μl magnetic beads suspension was used to detect the efficiency of immunoprecipitation. The protein on the magnetic beads was eluted with 1 × SDS-PAGE loading buffer and heated at 95 °C. Magnetic beads can be instantly submerged to the bottom of the tube, and supernatant can be directly used for SDS-PAGE.

### Statistical analysis

Statistical analyses were performed using GraphPad Prism 9.0 software (Prism 9: Taking your analyses and graphs to higher dimensions [graphpad.com]), and all data were presented as mean ± SEM. ∗*p* < 0.05, ∗∗*p* < 0.01, ∗∗∗*p* < 0.001, and ∗∗∗∗*p* < 0.0001 using Student’s *t* test. Statistical significance was assessed with two-way ANOVA with Dunnett’s multiple comparisons tests. All experiments were repeated at least three times.

## Data availability

The data that support the findings of this study are available from the corresponding author, Zhang Yanfen, upon reasonable request.

## Supporting information

This article contains supporting information (21).

## Conflict of interest

The authors declare that they have no conflicts of interest with the contents of this article.
