# Supplementary material for: Subcellular localization and function analysis of PINK1 mitron in PD progression: Mitron modulates mitochondrial morphology to regulate neuronal death
Source: J Biol Chem. 2024 Sep 12;300(10):107773. doi: 10.1016/j.jbc.2024.107773 (PMC11497375; doi:10.1016/j.jbc.2024.107773)
Supplement: Supporting Figures S1-S4 [file mmc1.docx]

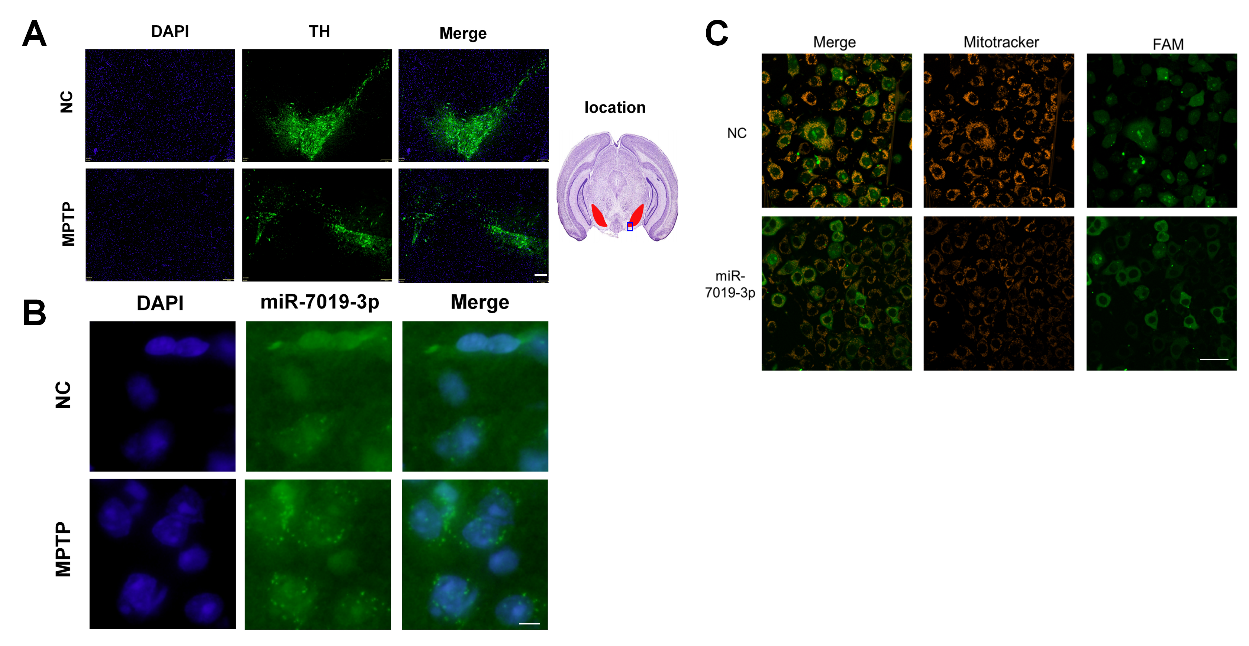


**Figure.S1** (A) Tyrosine hydroxylase (TH) was detected (green) in MPTP induced PD mice model, to identify the PD model. Scale bar: 150 μm. (B) In MPTP induced PD mice model, miR-7019-3p (green) exists in cytoplasm around the cell nucleus. Scale bar: 10 μm. (C) FAM modified synthetic miR-7019-3p were transfected into neuron cells, the fluorescence signals of exogenous transfection of miR-7019-3p were mainly found in the cytoplasm (green), and the fluorescence signals did not converge with the mitochondrial fluorescence signals(orange). Scale bar: 50 μm.


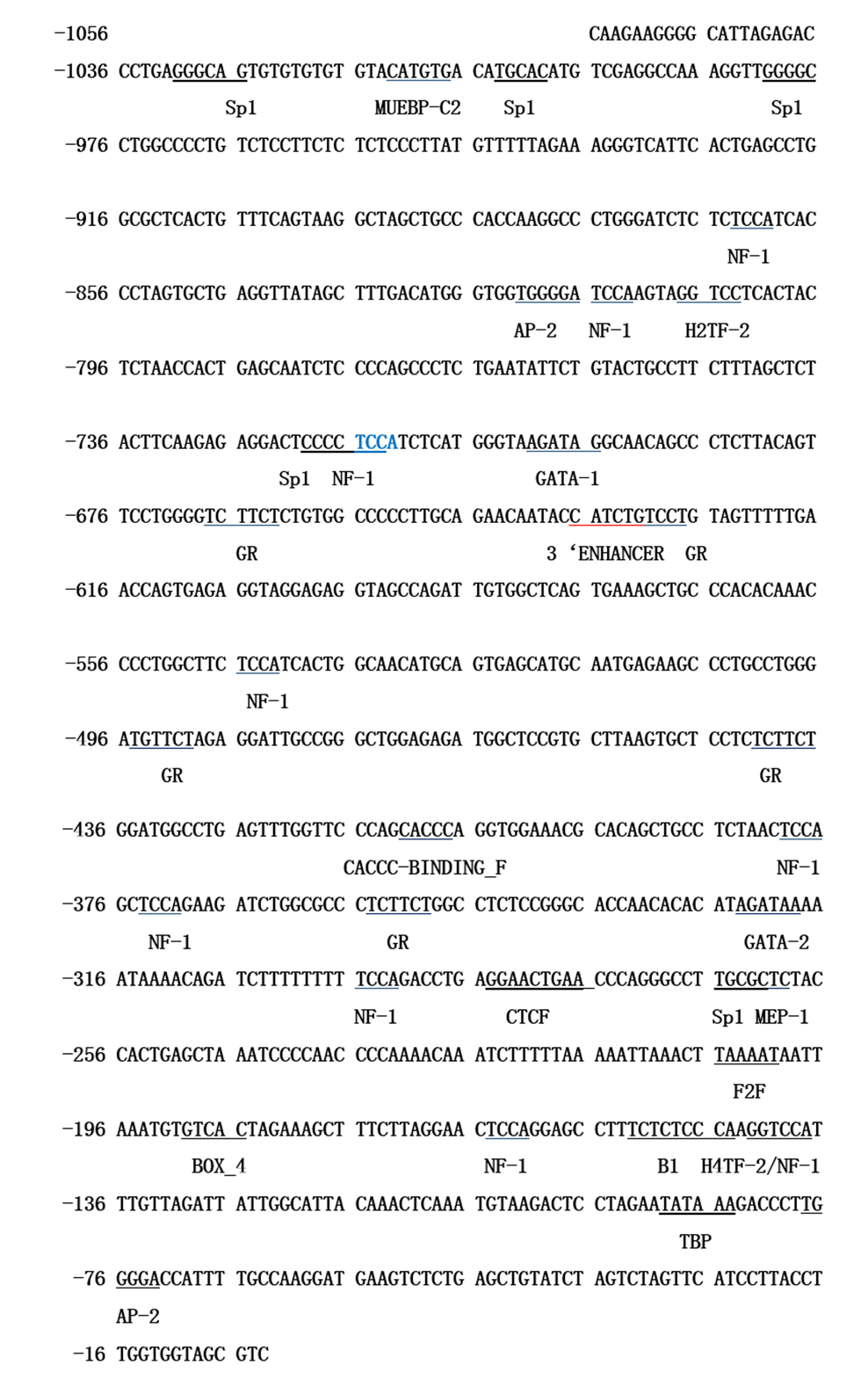


**Figure.S2** The predicted binding sequence of SP1 transcription factor on the upstream of miR-7019-3p encoded region.


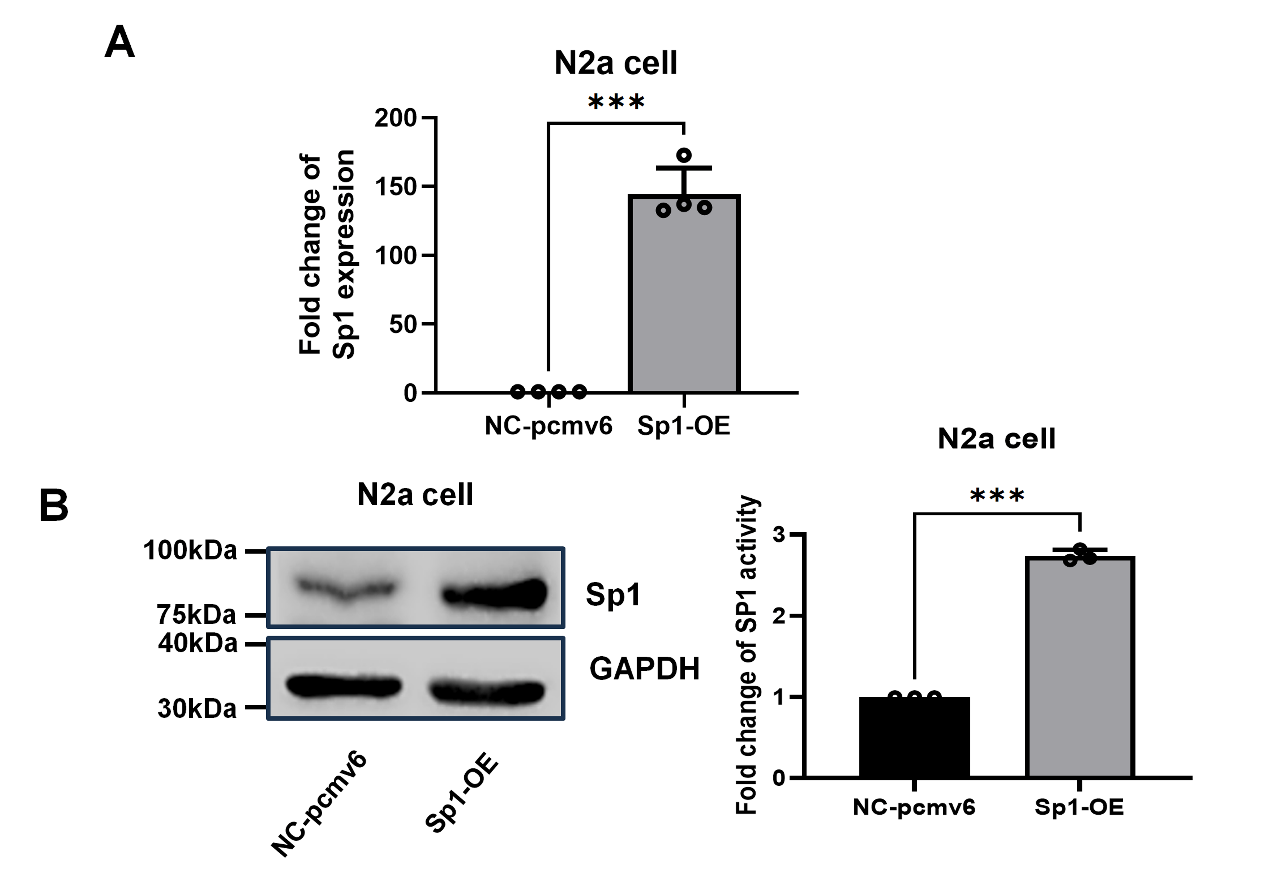


**Figure.S3** Sp1 was over expressed in N2a cells. (A) Sp1 mRNA expression in NC and over expression groups. (B) Sp1 protein levels in NC and over expression groups. *: p<0.05, ***: P<0.001.


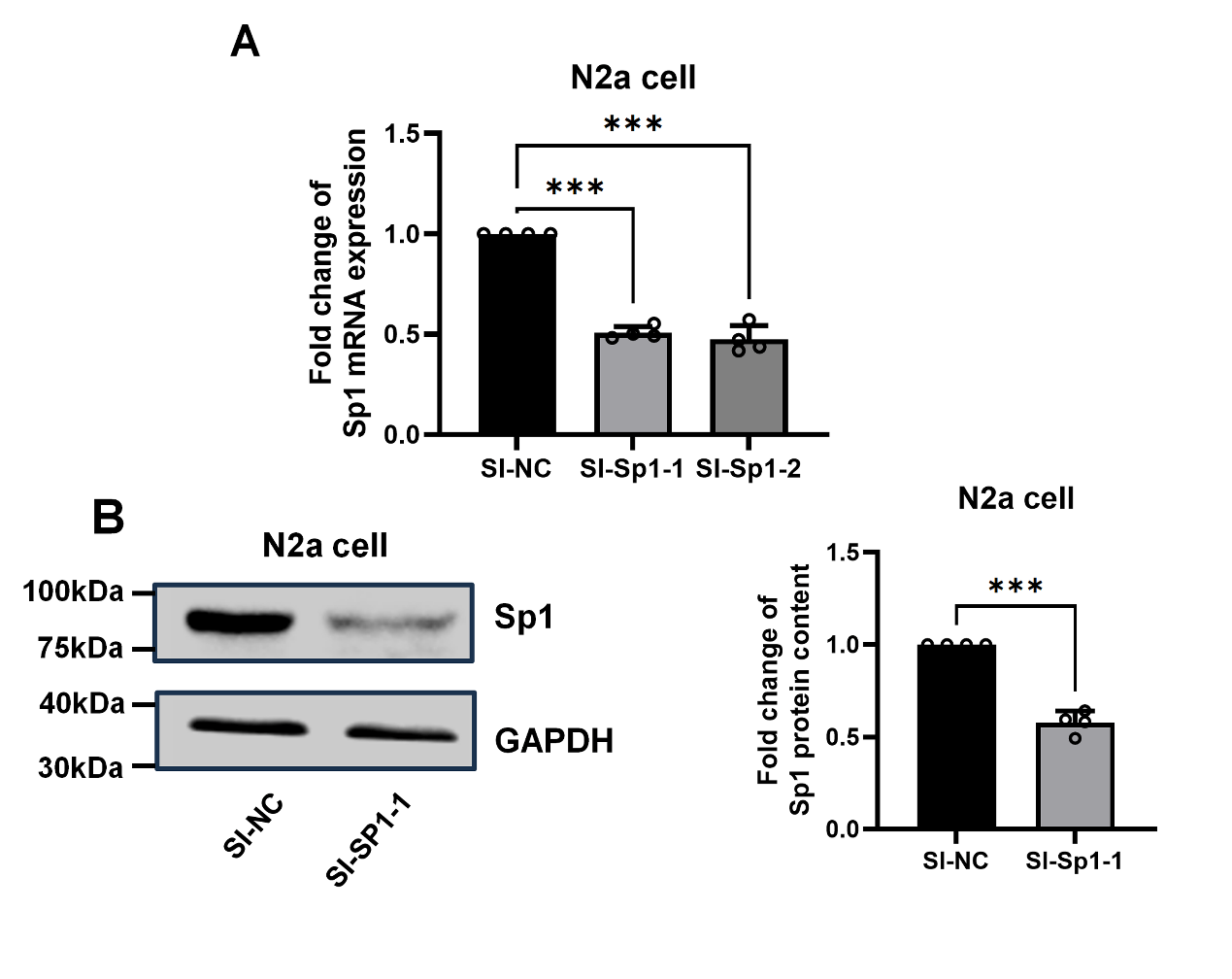


**Figure.S4** Sp1 mRNA level was knocked down with siRNA in N2a cells. (A) Sp1 mRNA expression in NC and knock down groups. (B) Sp1 protein levels in NC and knock down groups. *: p<0.05, **: p<0.01, ***: P<0.001.
